# WHO global research priorities for sexually transmitted infections

**DOI:** 10.1016/S2214-109X(24)00266-3

**Published:** 2024-07-20

**Authors:** Sami L Gottlieb, Erica Spielman, Laith Abu-Raddad, Adeniyi Kolade Aderoba, Laura H Bachmann, Karel Blondeel, Xiang-Sheng Chen, Tania Crucitti, Gabriela Garcia Camacho, Sheela Godbole, Rodolfo Gómez Ponce de Leon, Somesh Gupta, Joumana Hermez, Naoko Ishikawa, Jeffrey D Klausner, Firdavs Kurbonov, Ismael Maatouk, Ahmed Mandil, Maeve B Mello, Angelica Espinosa Miranda, Fausta Shakiwa Mosha, Joseph Chukwudi Okeibunor, Jason J Ong, Remco P H Peters, Freddy Pérez, Nicole Seguy, Kate L Seib, Mukta Sharma, Tim Sladden, Barbara Van Der Pol, Peter J White, Teodora Wi, Nathalie Broutet

**Affiliations:** a Department of Sexual and Reproductive Health and Research, WHO, Geneva, Switzerland; b Global HIV, Hepatitis, and STIs Programmes, WHO, Geneva, Switzerland; c Weill Cornell Medicine–Qatar, Doha, Qatar; d WHO Regional Office for Africa, Brazzaville, Republic of the Congo; e Wake Forest University School of Medicine, Winston-Salem, NC, USA; f National Center for STD Control, Nanjing, China; g Institut Pasteur de Madagascar, Antananarivo, Madagascar; h Indian Council of Medical Research–National AIDS Research Institute, Pune, India; i Latin American Center for Perinatology, Women's and Reproductive Health, Pan American Health Organization, Montevideo, Uruguay; j All India Institute of Medical Sciences, New Delhi, India; k WHO Regional Office for the Eastern Mediterranean, Cairo, Egypt; l Kawasaki Settlement Clinic, CFMD, Tokyo, Japan; m University of Southern California, Los Angeles, CA, USA; n High Institute of Public Health, University of Alexandria, Alexandria, Egypt; o Universidade Federal do Espírito Santo, Ministério da Saúde, Brasilia, Brazil; p Melbourne Sexual Health Centre, Melbourne, VIC, Australia; q Foundation for Professional Development, East London, South Africa; r Pan American Health Organization, Washington, DC, USA; s WHO Regional Office for Europe, Copenhagen, Denmark; t Institute for Glycomics, Griffith University, Gold Coast, QLD, Australia; u WHO Regional Office for South-East Asia, New Delhi, India; v UNFPA, New York, NY, USA; w University of Alabama at Birmingham Heersink School of Medicine, Birmingham, AL, USA; x Imperial College School of Public Health, London, UK; y UK Health Security Agency, London, UK

## Abstract

Sexually transmitted infections (STIs) are widespread worldwide and negatively affect sexual and reproductive health. Gaps in evidence and in available tools have long hindered STI programmes and policies, particularly in resource-limited settings. In 2022, WHO initiated a research prioritisation process to identify the most important STI research areas to address the global public health need. Using an adapted Child Health and Nutrition Research Initiative methodology including two global stakeholder surveys, the process identified 40 priority STI research needs. The top priorities centred on developing and implementing affordable, feasible, rapid point-of-care STI diagnostic tests and new treatments, especially for gonorrhoea, chlamydia, and syphilis; designing new multipurpose prevention technologies and vaccines for STIs; and collecting improved STI epidemiologic data on both infection and disease outcomes. The priorities also included innovative programmatic approaches, such as new STI communication and partner management strategies. An additional six research areas related to mpox (formerly known as monkeypox) reflect the need for STI-related research during disease outbreaks where sexual transmission can have a key role. These STI research priorities provide a call to action for focus, investment, and innovation to address existing roadblocks in STI prevention, control, and management to advance sexual and reproductive health and wellbeing for all.

## Introduction

Sexually transmitted infections (STIs) have a profound effect on sexual and reproductive health worldwide. More than one million STIs are acquired every day.^1^ In 2020, an estimated 374 million new infections occurred with one of four bacterial or parasitic STIs that can be cured with available antimicrobials: *Treponema pallidum* (syphilis), *Chlamydia trachomatis* (chlamydia), *Neisseria gonorrhoeae* (gonorrhoea), and *Trichomonas vaginalis* (trichomoniasis).^1^ Additionally, viral STIs that can be long lasting (eg, human papillomavirus [HPV]) or lifelong (eg, herpes simplex virus) affect hundreds of millions of people worldwide at any point in time.^2^ The burden of STIs is greatest in low-income and middle-income countries (LMICs).^1^, ^2^ In addition to genital symptoms, STIs can lead to multiple adverse sexual and reproductive health outcomes, including cervical cancer, infertility, increased vulnerability to HIV, pregnancy complications, and congenital infections. Moreover, the psychological, social, and economic consequences of STIs can substantially affect quality of life.

Despite the need, the public health response to STIs has been poor in many parts of the world. As the WHO global strategy on STIs for 2016–21 came to a close,^3^ WHO issued a global progress report on the strategy, noting that STI control had shown little improvement.^1^ Long-standing challenges, such as the absence of available STI diagnostic tests in resource-limited settings, combined with newer threats, such as global shortages of benzathine penicillin for the treatment of syphilis, increasing gonococcal antimicrobial resistance, and outbreaks of sexually transmissible viruses (eg, Ebola virus and monkeypox virus), have hampered STI control.^4^, ^5^, ^6^ The WHO global progress report^1^ called for a coordinated research agenda to address gaps in knowledge and in availability of tools, which have hindered advances in STI prevention, control, and management, particularly in resource-limited settings.

Following this call, the new WHO global strategy on STIs for 2022–30 highlighted research and innovation as a fundamental component of the global response.^7^ In 2022, WHO initiated a global STI research priority setting exercise to identify the most crucial research areas to address the global public health need. The exercise aimed to: (1) develop global STI research priorities reflecting input from diverse experts and stakeholders across all WHO geographical regions; (2) provide considerations for adapting the global research priorities to regional and setting-specific contexts; and (3) disseminate the research priorities widely, offering direction and guidance for research on STIs until 2030. Future STI research based on these priorities will contribute substantially to achieving the Sustainable Development Goals for 2030, specifically ensuring healthy lives and promoting wellbeing for all, through ensuring universal access to sexual and reproductive health-care services and combating communicable diseases.^8^

This Health Policy paper presents findings from the global STI research prioritisation exercise. It is intended to inform researchers, funding institutions, policy makers, implementing partners, industry, and civil society on the research areas that will address the most important evidence gaps and develop the most needed interventions to reduce the global toll of STIs.

## Methods

An STI research prioritisation working group was established to lead the prioritisation process (appendix p 2). It consisted of two subgroups: the WHO secretariat, including seven representatives from WHO headquarters and eight from WHO regional offices, and the STI Research Priority Setting Technical Advisory Group, comprised of 16 external experts competitively selected by the secretariat through an open call for applications according to standard WHO procedures,^9^ considering areas of expertise, geographical representation, and potential conflicts of interest.

**Figure 1:**
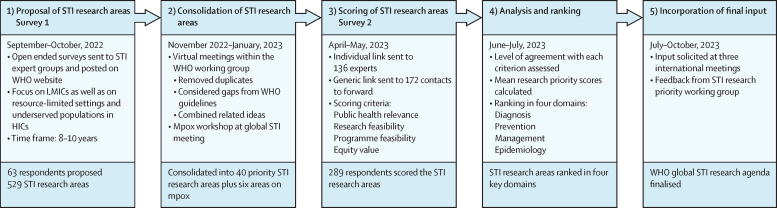
The five phases of the STI research priority setting process HICs=high-income countries. LMIC=low-income and middle-income countries. STI=sexually transmitted infection.

The working group followed WHO guidance on a systematic approach for global research priority setting exercises^10^ to develop a protocol for the STI research prioritisation process. The protocol used the Child Health and Nutrition Research Initiative (CHNRI) methodology^11^, ^12^ outlined in five phases to: propose, consolidate, score, analyse and rank, and finalise the priority STI research areas (figure 1). The CHNRI methodology allows flexibility for adapting to different questions and contexts, and thus adaptations were made in choosing the scoring criteria most relevant for global STI research and simplifying the ranking process within key STI domains.^11^, ^12^

The research priority setting process aligned with the WHO Global Health Sector Strategy on STIs,^7^ including a focus on LMICs as well as on resource-limited settings and underserved populations in high-income countries (HICs), over a timeframe of 8–10 years. Research priorities related to HIV, hepatitis, HPV vaccination, and cervical cancer prevention were not considered in the current priority setting process, as they are addressed elsewhere.^13^

The protocol was evaluated by the WHO Ethics Review Committee and received a research exemption (WHO/ERC ERC.0003782). Implementation of the priority setting process took place between September, 2022 and October, 2023.

### Proposal of STI research areas

The first phase of the process was to generate STI research ideas from surveying a broad range of STI experts and stakeholders (survey 1; appendix p 4). Survey 1 was published online and disseminated directly using established WHO lists of STI experts from STI guideline development groups and other WHO advisers. The survey was also advertised during the International Union Against STIs (IUSTI) World Congress in Zimbabwe on Sept 4–7, 2022.

STI experts and stakeholders were sent emails encouraging them to participate and share the links with others. This snowball method was used to reach a broad range of participants.

To avoid leading participants' responses, priority STI research areas were solicited according to three generic categories of research, as recommended for the CHNRI process:^12^ (1) research to understand the extent of the problem and its predictors (eg, epidemiology, risk factors, and disease burden); (2) research to develop and evaluate new interventions (eg, technologies, products, programmes, and clinical strategies); and (3) research to implement existing STI interventions (eg, acceptability, feasibility, effectiveness, or cost-effectiveness).

Participants could also propose additional research areas outside of these categories. For each category, participants were instructed to propose up to three to five research areas involving any STI pathogens, populations, or settings they perceived to be important.

The survey was prepared in English and translated into Arabic, Chinese, French, Russian, Portuguese and Spanish. Online survey data were captured using OpenClinica.

### Consolidation of STI research areas

Through a series of virtual meetings, the working group consolidated the STI research areas proposed in survey 1 by removing duplicates, combining related areas, and further refining language. To fill any major gaps, the working group also considered STI research needs identified during the development of WHO STI guidance documents.

Consolidation of STI research areas related to mpox (formerly known as monkeypox) followed a slightly different process. Planning was underway for survey 1 when a multicountry outbreak of mpox, driven largely by sexual transmission, was declared a public health emergency of international concern by WHO.^14^ In September, 2022, WHO convened a symposium at the IUSTI World Congress to discuss research questions related to sexual transmission of monkeypox virus. The outcomes of these discussions were combined with mpox-related research areas from survey 1 to form a separate list. The list was further refined with input from the WHO secretariat of the International Health Regulations Emergency Committee on the Multi-Country Outbreak of Monkeypox.^15^

### Scoring of STI research areas

A second online survey (survey 2; appendix p 11) was conducted asking participants to score each of the consolidated STI research areas based on how strongly they agreed with four predefined criteria: (1) public health relevance—the emerging intervention is likely to substantially improve health; (2) research feasibility—it will be possible to design an ethically sound and implementable research study to address the area; (3) programme feasibility—the research results can be translated into a deliverable and affordable public health intervention; and (4) equity value—addressing the research area can facilitate interventions that reduce population inequities.

Scoring used a Likert scale: 5 for strongly agree, 4 for agree, 3 for neutral, 2 for disagree, and 1 for strongly disagree. Participants randomly received one of six survey versions that rotated the order of the research areas to avoid fatigue bias.

Survey 2 was sent through individual links to 136 STI experts recommended by the working group to ensure adequate representation according to geography and expertise. In addition, a generic survey link was sent to 172 contacts representing leaders of STI-related research, health-care professionals, public health officials, civil society, and implementing organisations and additional stakeholders from established WHO lists, as per survey 1. Survey 2 was posted on the WHO website and shared through email lists, newsletters, and social media. Individuals receiving the generic survey link were asked to share it widely.

Survey 2 was translated into French, Portuguese, and Spanish. Data were collected using REDCap.

### Analysis and ranking

Frequencies, median, and range values were calculated, as appropriate, for data collected on gender identity, age group, WHO regions and country-income levels of the primary focus of the respondent's work, number of years of experience related to STIs, main fields of expertise, primary occupation, and main type of employer or affiliated organisation.

Analysis of STI research priority area scoring from survey 2 was conducted in two ways: (1) mean research priority score:^11^ for each research area, the Likert scores were summed across all four criteria for each participant; the sum was then divided by the maximum score possible (ie, 5 for each criterion; 20 if all four criteria were scored), resulting in a research priority score out of 100; the four criteria were weighted equally; the mean research priority score was calculated by adding the summary scores across participants and dividing by the number of respondents scoring the area and (2) the percentages of respondents reporting they strongly agreed, agreed, were neutral, disagreed, or strongly disagreed with each of the scoring criteria (public health relevance, research feasibility, programme feasibility, and equity value) were also calculated.

Sub-analyses of the STI research priority areas were performed by WHO regions (Africa, the Americas, South-East Asia, Europe, Eastern Mediterranean, Western Pacific) and country-income levels (LMICs, HICs, or both) according to the World Bank classification.

STI research areas were ranked according to mean research priority scores.

### Incorporation of final input

The CHNRI method allows for different ways of gathering further input on drafting research priority lists. The preliminary results were discussed within the working group and at three international meetings: the Women's Health Innovation Equity Forum, Washington DC, USA, July, 2023;^16^ the WHO symposium on the global STI research priorities at the STI & HIV 2023 World Congress, Chicago, IL, USA, July, 2023; and the World Health Summit 2023 post-conference WHO meeting: Mobilize for action to address sexually transmitted infections!, Berlin, Germany, October, 2023. Input from these meetings helped shape the interpretation of the results in this paper.

### Role of the funding source

WHO supported and coordinated this work. WHO staff were involved in the design of the research prioritisation process, in data collection, analysis, and interpretation, and in the writing of the report.

## Findings

### Proposal of STI research areas

61 respondents completed online survey 1 and two respondents sent their proposed STI research areas via email (figure 1). Of the 61 online respondents, 35 identified themselves as women, 25 as men, and one as non-binary. Approximately half of respondents were aged 45 years or older. Respondents were geographically diverse, and 51 (84%) reported working in LMICs. 46 respondents (75%) reported more than one area of expertise.

The 63 STI experts and stakeholders generated 529 STI research areas: 194 areas related to understanding the extent of the problem, 144 related to developing and evaluating new interventions, 128 related to implementing existing interventions, and 63 additional areas not addressed in the preselected categories.

### Consolidation of STI research areas

The 529 proposed STI research areas greatly overlapped or addressed similar topics, and after removing duplicates and combining related areas, the working group consolidated them into 40 priority STI research areas (11 on research to understand the problem, 19 on research to develop new interventions, and ten on research to implement existing interventions). Six additional research areas were related to sexually acquired mpox. For the precise wording of the 46 areas, see survey 2 in the appendix (p 11).

### Respondents to the scoring survey

Of the 136 STI experts and stakeholders receiving individual links to survey 2, 64 (47%) completed the scoring survey. Another 225 respondents completed the survey through the generic link, resulting in a total of 289 completed surveys (figure 1). Of the 287 respondents with available gender data, 151 (53%) identified themselves as women, 133 (46%) as men, and three identified as non-binary or preferred not to describe their gender (appendix p 16). Respondents represented all WHO regions and, of 284 respondents with available data, 61 (21%) reported working in more than one WHO region or globally, 56 (20%) in the South-East Asian region, 55 (19%) in the region of the Americas, and 37 (13%) in the African region. The majority reported working primarily in LMICs (210 [73%] of 287 with available data) or in both LMICs and HICs (31 [11%] of 287).

Of 289 respondents, 111 (38%) were health-care providers, 73 (25%) researchers, 43 (15%) programme implementers and policy makers, 34 (12%) academics, and 28 (10%) represented other occupations (appendix p 16). Respondents were mainly employed by academic or research institutions (87, 30%), national or regional government (66, 23%), and hospitals or clinics (46, 16%). The majority (210, 73%) of respondents reported more than one area of expertise: 201 (70%) reported expertise on STIs other than HIV, 150 (52%) on HIV, 109 (38%) on infectious disease, and 106 (37%) on sexual and reproductive health.

### Scoring and ranking of STI research areas

Mean research priority scores for the 40 STI research areas ranged from 76 to 91, showing that all were considered relatively high priority. Consequently, for clarity and ease of presentation, the working group decided to organise the STI research areas, ranked by mean research priority score, within four main domains of STI research: diagnosis (mean score 86, range 78–91), prevention (85, 83–87), management (84, 79–89), and epidemiology (83, 76–88).

Scoring results for the STI research areas in each domain and ranking of STI research priorities within each domain, overall and broken down by WHO region and by income level, are presented in the appendix (pp 18–25).

**Figure 2:**
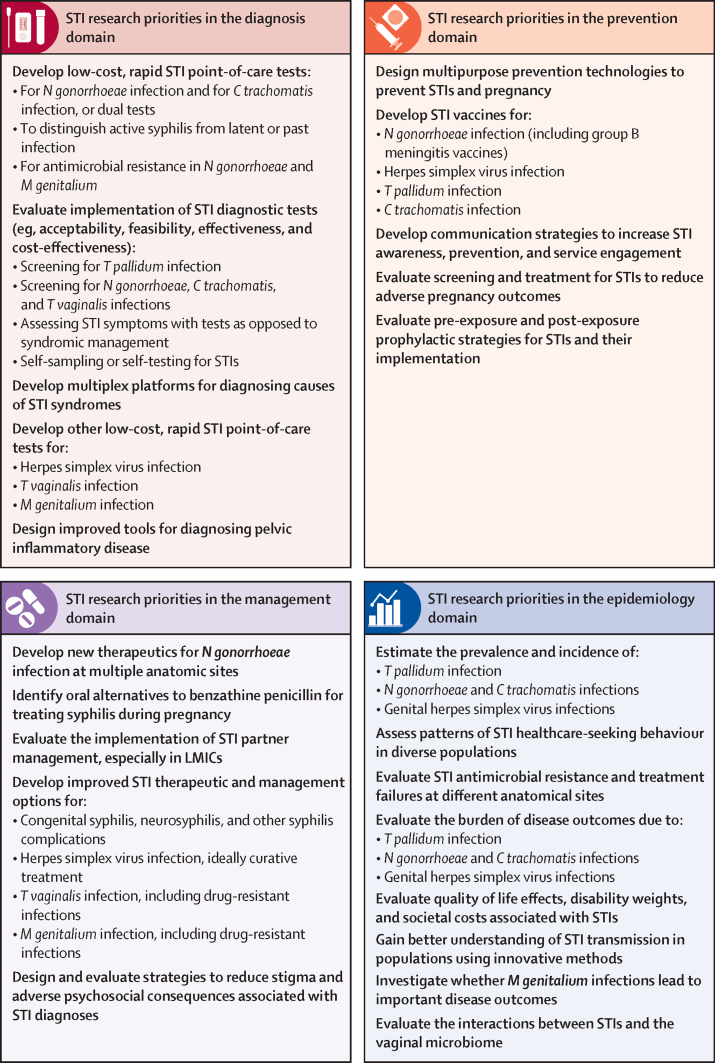
Summary of the global STI research priorities by research domain *C trachomatis*=*Chlamydia trachomatis* (chlamydia). LMICs=low-income and middle-income countries. *M genitalium=Mycoplasma genitalium. N gonorrhoeae*=*Neisseria gonorrhoeae* (gonorrhoea). STI=sexually transmitted infection. *T pallidum=Treponema pallidum* (syphilis). *T vaginalis=Trichomonas vaginalis*.

Figure 2 summarises the priority STI research areas by domain, with some grouping of related items with similar research priority scores.

In the diagnosis domain (figure 2; appendix p 18), the top four STI research areas were related to development and implementation of low-cost, rapid point-of-care tests for gonorrhoea, chlamydia, and syphilis, which showed high levels of agreement across all four public health criteria. These research areas were in the top priorities across geographical regions, and for both LMICs and HICs (appendix p 22). Research on use of rapid diagnostics for STI symptoms as opposed to syndromic management was a slightly higher priority for LMICs and the African and South-East Asian regions, whereas research on STI self-sampling or self-testing was a higher focus in HICs.

In the STI prevention domain (figure 2; appendix p 19), a different scoring pattern was observed. A high proportion of respondents strongly agreed that development of several new STI prevention interventions has high public health relevance. However, research feasibility had lower overall agreement. Multipurpose prevention technologies and STI vaccines had the largest disconnect between perceived public health relevance and research feasibility. Ranking varied by region and country-income level (appendix p 23). Multipurpose prevention technologies for STIs and pregnancy were a more prominent focus of stakeholders working in LMICs, whereas STI vaccine development was a greater focus in HICs.

In the STI management domain (figure 2; appendix p 20), two research areas consistently scored highest, including across regions and income levels—identification of new therapies for drug-resistant *N gonorrhoeae* and oral alternatives to benzathine penicillin G for treatment of syphilis in pregnancy. Although most research areas in this domain related to therapeutics, other key areas included research on STI partner management, particularly in the African and Western Pacific regions and LMICs (appendix p 24). Strategies to reduce stigma and psychosocial consequences of STIs was more prominent in the Eastern Mediterranean region and in HICs.

In the epidemiology domain (figure 2; appendix p 21), 12 STI research areas were evaluated. Research to estimate syphilis prevalence, incidence, and disease burden and patterns of STI health-care-seeking behaviour were prominent, as were the epidemiology of antimicrobial resistance and the infection and disease burden related to gonococcal and chlamydial infections. Some regions were more focused on particular pathogens; eg, burden of disease due to gonorrhoea and chlamydia in the African region and due to syphilis in the Americas (appendix p 25).

### Research priorities for sexually acquired mpox

The six research areas related to sexually acquired mpox are shown in the appendix (p 27). Mean research priority scores ranged between 75 and 80. The highest scoring areas were related to effectiveness of vaccines and antiviral treatments, and barriers to prevention and care and effective risk communication strategies for sexually acquired mpox. Research related to monkeypox virus natural history, transmission dynamics, and determinants of acquisition followed close behind.

## Discussion

Using a systematic and comprehensive approach, the WHO global STI research prioritisation process identified 40 important STI research areas that would address the greatest gaps in STI guidance, policies, and programmes worldwide. The process focused on LMICs as well as resource-limited settings and underserved populations in HICs, which are disproportionately affected by STIs.^1^ The top priorities centred around developing and implementing affordable, feasible rapid point-of-care diagnostics and treatment interventions for gonorrhoea, chlamydia, and syphilis; designing new prevention interventions for these curable STIs as well as herpes simplex virus infection; and collecting improved STI epidemiologic data on both infection and disease outcomes. The research priorities also included innovative programmatic approaches, such as new STI communication and management strategies. An additional six research areas were identified related to mpox, reflecting the need for STI-related research in the context of disease outbreaks, where sexual transmission can have a key role.

Development of STI point-of-care diagnostic tests, such as to detect *N gonorrhoeae and C trachomatis* infections and to distinguish active syphilis from latent or previous infection, and research on their implementation, were considered to have both high public health relevance and high feasibility, reflecting a much needed quick win. In much of the world, STI programmes rely on syndromic management, using collections of symptoms to guide diagnosis and treatment. However, most STIs are asymptomatic and thus missed.^5^, ^17^ New diagnostics are crucial for delivering quality STI services, reducing the spread of STIs, and improving data collection.^18^

New STI prevention interventions, such as multipurpose prevention technologies that prevent both STIs and pregnancy as well as new STI vaccines such as those against *N gonorrhoeae* infection, are a priority for long-term, sustainable STI control.^19^, ^20^ Experts considered research on new STI prevention interventions to have high public health relevance but lower research feasibility in the short term. It is unclear whether the lower perceived feasibility was related to difficulty in developing the interventions, the size or expense of conducting clinical trials, or the longer perceived timeframe for development. A longer timeline is likely realistic for interventions such as syphilis and chlamydial vaccines, which are relatively early in development.^19^ Further addressing these development barriers will be crucial to advancing these important interventions.^19^, ^20^

New STI therapeutics can address some immediate challenges to STI control. New therapies for gonorrhoea are important, given concerning increases in antimicrobial resistance against extended spectrum cephalosporins, the last remaining first-line therapy for gonococcal infection.^4^ Alternatives to benzathine penicillin G for treating syphilis, including syphilis in pregnancy, are greatly needed, given recurrent global shortages.^6^ New management options are also needed for congenital syphilis. Like the biomedical prevention interventions, programmatic innovations in managing partners of people with STIs were perceived by experts to have high public health relevance but lower research and programmatic feasibility.

Improved STI epidemiologic data and estimates of infection and disease burden underpin all the other efforts and are essential. Such data can help raise awareness about STIs, target prevention and control efforts to areas or populations most in need, monitor the impact of existing interventions, and provide the basis for understanding the full potential value, and thus investment, in new STI interventions. Experts recognised that not only are better infection estimates needed in different settings, but also estimates of the burden of disease due to STIs, such as infertility and other reproductive tract complications of chlamydia and gonorrhoea.^21^

The CHNRI method is not meant to be fully prescriptive, rather to show the collective optimism of a reasonably large group of experts on how multiple research areas would satisfy important public health criteria.^12^ The narrow range of research priority scores (ie, 76–91 of 100) in the scoring survey shows that all 40 STI research areas identified through the initial phases of the process were considered relatively high priority. Although certain areas were clearly highest priority, STI research areas within the lower end of the range are nonetheless important for global public health and could be key components of higher-ranked areas. For example, new tools for diagnosing pelvic inflammatory disease might be crucial in understanding the burden of disease due to chlamydial and gonococcal infection. It was somewhat surprising that the vaginal microbiome, bacterial vaginosis and other dysbiosis, and related prevention and treatment measures were not included among the priority areas, other than one area on the association between STIs and the vaginal microbiome. This may have been because experts felt this was out of scope for this exercise on STIs; however, this important area is included in a separate women's health research agenda.^16^, ^22^

A strength of the WHO STI research prioritisation process was its comprehensive and global nature and use of the CHNRI methodology adapted for the STI context. This standardised and transparent methodology has become the dominant approach to setting health priorities in the global biomedical literature over the past decade.^12^, ^23^ This Health Policy paper has also described the prioritisation process in alignment with the reporting guideline for priority setting of health research (known as REPRISE) framework.^24^ Representativeness of stakeholder groups is not a prerequisite for the methodology used.^11^, ^12^ Nonetheless, the experts involved were gender-balanced and represented all WHO regions, and the majority reported working in LMICs, a major focus of the STI research priorities. Survey 1 was translated into six languages to reach a broad range of stakeholders, and although no responses were received in Arabic, Chinese, or Russian, STI experts from all these linguistic regions were represented, completing the surveys in English or French. Furthermore, although previous research on the CHNRI method has shown that rankings of research ideas by independent experts become stable after about 40 participants,^23^, ^25^ many stakeholders (n=289) scored the STI research areas in survey 2, well beyond many previous CHNRI prioritisation exercises.^23^

Nonetheless, some limitations need to be considered. First, in any research prioritisation process, scoring can be affected by ongoing research in which self-selected participants have relevant interests. To minimise this bias, feedback was solicited from a broad range of stakeholders beyond researchers, who represented 25% of survey 2 respondents. Second, only 63 stakeholders participated in survey 1, potentially limiting the number of research areas proposed. However, the power of the proposal phase of the CHNRI method is not linked to the number of people participating but rather reaching a point of repetition; these 63 individuals generated 529 research areas that greatly overlapped. And third, potential overlap in those completing survey 1 and survey 2 could have resulted in some confirmation bias, with those proposing research areas also ranking them. A far greater number of stakeholders (n=289) participated in survey 2 to score the STI research areas, so even if all individuals completing survey 1 also completed survey 2, the influence of this bias would be relatively small.

The top STI research priorities were generally consistent across geographical regions and country-income status, and most respondents agreed that addressing these research areas would have public health importance. Priority research areas for sexually acquired mpox were similar to those for other STIs, but with a greater focus on infectiousness and transmission dynamics, natural history, and predictors of clinical presentation, which are especially pertinent for emerging infections and in outbreak contexts. Recent Ebola virus outbreaks showed that sexual transmissibility can be important, even for pathogens with other primary modes of transmission.^26^ Such research priorities need to be considered in advance for future outbreaks.

Elaboration of these STI research priorities outlines an important global research agenda and is a crucial first step. To implement this agenda, vital next steps include delineating the component activities for each research area, mapping currently funded or planned activities and remaining gaps, and outlining a roadmap to address the most critical research activity gaps. Individual regions or countries can further adapt the global STI research priorities to build a research portfolio of pertinent activities according to setting-specific contexts, needs, and potential impacts. It is of utmost importance that the interventions resulting from the priority STI research areas translate into actionable STI policies and programmes and reach the populations and settings that need them the most.^7^, ^8^

Successful implementation of the global STI research agenda will require widespread dissemination and efforts to increase political will and community advocacy related to STIs, potentially building on lessons learned from the HIV field.^27^ The COVID-19 pandemic catalysed several technological and health systems innovations that can be leveraged to advance the STI field, including new platforms and pathways for diagnostics and vaccines, expanded health information systems, and self-care approaches.^28^, ^29^ Advances in artificial intelligence and digital health solutions also provide opportunities for accelerating STI innovation.^30^ Dovetailing with related global research prioritisation initiatives, such as the Women's Health Innovation Opportunity Map 2023,^16^ can broaden possibilities for funding and focus.

## Conclusions and future directions

These STI research priorities provide a call to action for focus, investment, and innovation to address the global epidemic of STIs. For decades, STI control has been based on the same tools, and the world is far from reaching the 2030 goals of the WHO global strategy on STIs.^7^ Innovative approaches are needed. Without research and investment, STIs will continue to have a negative impact on the sexual and reproductive health of people worldwide, especially women and neonates, adolescents and young people, people living in LMICs, and marginalised populations in all settings. This WHO agenda should encourage researchers to focus on these priority STI research areas and donors to support this large research effort, in order to reach global goals toward ending STIs as a public health problem and advancing sexual and reproductive health and wellbeing for all.

### Contributors

## Declaration of interests

SLG received support from the Bill & Melinda Gates Foundation paid to WHO for work on therapeutic human papillomavirus vaccines and on COVID-19 vaccines and pregnancy, and the Gates Foundation travel support to meetings on maternal immunisation and women's health innovations. LHB received royalties for book editing on *Sexually Transmitted Infections in HIV-infected Adults and Special Populations*. JDK received grants and support from the US National Institutes of Health (NIH), US Centers for Disease Control and Prevention, and Open Philanthropy; royalties and licences from UpToDate and McGraw-Hill; one-time consulting fees from Visby Medical, Diagnostics Direct, and Biofire; payment or honoraria from AIDS Healthcare Foundation; and payment for expert testimony from Gray Robinson. He is currently President of the non-profit organisation Herpes Cure Advocacy. JJO received grant support paid to his institution from the Australian National Health and Medical Research Council (NHMRC) and US NIH; and honoraria for presentations or other speaking events and support for attending meetings from Gilead Sciences. He is currently Board Director for the Australian Society for HIV, Hepatitis and Sexual Health Medicine and for Health Equity Matters and is a World Executive Committee member for the International Union against Sexually Transmitted Infections (IUSTI). RPHP received grants from NIH, Global AMR Innovation Fund–Foundation for Innovative New Diagnostics, Open Philanthropy, Global Antibiotic Research and Development Program–South African Medical Research Council, and the Swiss National Science Foundation**.** KLS received grants paid to her institute from the Australian NHMRC (numbers 1182443, 2017383, and 2002182). She has also received airfare and conference registration support from the International Pathogenic Neisseria Conference and the IUSTI World Congress. PJW received support from the UK Medical Research Council and National Institute for Health and Care Research, salary from the UK Health Security Agency and Imperial College London, and consulting fees from Pfizer and the National Institute for Public Health and the Environment. BVDP received consulting fees as an advisory board member for Abbot Rapid Diagnostics and Detect, consulting fees from Preventx, and speaking honoraria from Roche. She is President of the International Society for STD Research. NB received a consultancy contract from WHO for assistance with this project and had a contract with the Daffodil Centre on cervical cancer elimination. All other authors report no competing interests.
