# Supplementary material for: WHO global research priorities for sexually transmitted infections
Source: Lancet Glob Health. 2024 Jul 20;12(9):e1544–51. doi: 10.1016/S2214-109X(24)00266-3 (PMC11342064; doi:10.1016/S2214-109X(24)00266-3)
Supplement: Supplementary appendix [file mmc1.pdf]

THE LANCET
Global Health

Supplementary appendix

This appendix formed part of the original submission and has been peer reviewed. We post it as supplied by the authors.

Supplement to: Gottlieb SL, Spielman E, Abu-Raddad L, et al. WHO global research priorities for sexually transmitted infections. *Lancet Glob Health* 2024; published online July 20. https://doi.org/10.1016/S2214-109X(24)00266-3.

Supplementary appendix: WHO global research priorities for sexually transmitted infections

Table of Contents

The WHO STI Research Prioritization Working Group 2

SURVEY 1 – Proposal of STI Research Priorities 4

SURVEY 2 – Scoring of STI Research Priorities........ 11

Table S1. Demographics of the respondents to the STI research priority scoring survey 16

Figure S1. Priority STI research areas within the diagnosis domain, according to levels of stakeholder agreement and mean research priority score 18

Figure S2. Priority STI research areas within the prevention domain, according to levels of stakeholder agreement and mean research priority score 19

Figure S3. Priority STI research areas within the management domain, according to levels of stakeholder agreement and mean research priority score 20

Figure S4. Priority STI research areas within the epidemiology domain, according to levels of stakeholder agreement and mean research priority score........ 21

Table S2. Diagnosis domain: STI research areas ranked by research priority score (RPS), overall and by WHO Region and by country-income level........ 22

Table S3. Prevention domain: STI research areas ranked by research priority score (RPS), overall and by WHO Region and by country-income level........ 23

Table S4. Management domain: STI research areas ranked by research priority score (RPS), overall and by WHO Region and by country-income level........ 24

Table S5: Epidemiology domain: STI research areas ranked by research priority score (RPS), overall and by WHO Region and by country-income level........ 25

Table S6. Research priorities related to sexually acquired mpox (monkeypox virus) 27

The WHO STI Research Prioritization Working Group

The World Health Organization (WHO) STI Research Priority Setting Working Group consisted of two groups:

1) the WHO secretariat, including seven representatives from WHO headquarters and eight from WHO Regional Offices, and
2) the WHO STI Research Priority Setting Technical Advisory Group (TAG), comprised of 16 external experts competitively selected by the secretariat through an open call for applications according to standard WHO procedures,[1] considering areas of expertise, geographic representation, and potential conflicts of interest.

WHO Secretariat

**Name**	**Gender**	**Affiliation**	**Country**	**WHO Region**
Karel Blondeel	M	WHO Department of Sexual and Reproductive Health and Research (SRH) and Global HIV, Hepatitis and STIs Programmes (HHS)	Switzerland	Headquarters (HQ)
Nathalie Broutet	F	WHO SRH	Switzerland	HQ
Rodolfo Gómez Ponce de Leon	M	Latin American Center for Perinatology, Women's and Reproductive Health (CLAP/WR), Pan American Health Organization (PAHO) (WHO Regional Office of the Americas [AMRO])	Uruguay	AMRO
Sami Gottlieb	F	WHO SRH	Switzerland	HQ
Joumana Hermez	F	WHO Regional Office for the Eastern Mediterranean (EMRO)	Egypt	EMRO
Ismael Maatouk	M	WHO HHS	Switzerland	HQ
Ahmed Mandil	M	WHO EMRO and High Institute of Public Health, University of Alexandria	Egypt	EMRO
Maeve B. Mello	F	WHO HHS	Switzerland	HQ
Fausta Shakiwa Mosha	F	WHO Regional Office for Africa (AFRO)	Republic of Congo	AFRO
Joseph Chukwudi Okeibunor	M	WHO AFRO	Republic of Congo	AFRO
Freddy Pérez	M	WHO PAHO	USA	AMRO
Nicole Seguy	F	WHO Regional Office for Europe (EURO)	Denmark	EURO
Mukta Sharma	F	WHO Regional Office for South-East Asia (SEARO)	India	SEARO
Erica Spielman	F	WHO SRH and HHS	Switzerland	HQ
Teodora Wi	F	WHO HHS	Switzerland	HQ

WHO STI Research Priority Setting Technical Advisory Group

**Name**	**Gender**	**Affiliation**	**Country**	**WHO Region**
Laith Abu-Raddad	M	Weill Cornell Medicine-Qatar	Qatar	EMRO
Adeniyi Aderoba	M	Consultant obstetrician-gynaecologist	Nigeria and UK	AFRO and EURO
Xiang-Sheng Chen	M	National Center for STD Control	China	WPRO
Laura Bachmann	F	Wake Forest University School of Medicine	USA	AMRO
Tania Crucitti	F	Institut Pasteur de Madagascar	Madagascar and Belgium	AFRO and EURO
Sheela Godbole	F	Indian Council of Medical Research – National AIDS Research Institute	India	SEARO

[1] https://www.who.int/about/collaboration/open-calls-for-advisory-groups

Somesh Gupta	M	All India Institute of Medical Sciences	India	SEARO
Naoko Ishikawa	F	Kawasaki Settlement Clinic	Japan	WPRO
Jeffrey D. Klausner	M	University of Southern California	USA	AMRO
Angelica Espinosa Miranda	F	Universidade Federal do Espírito Santo (UFES), Ministério da Saúde	Brazil	AMRO
Jason Ong	M	Melbourne Sexual Health Centre	Australia	WPRO
Remco Peters	M	Foundation for Professional Development	South Africa	AFRO
Kate Seib	F	Institute for Glycomics, Griffith University	Australia	WPRO
Tim Sladden	M	UNFPA	Thailand and Australia	SEARO and WPRO
Barbara Van Der Pol	F	University of Alabama at Birmingham Heersink School of Medicine	USA	AMRO
Peter White	M	Imperial College School of Public Health and UK Health Security Agency	UK	EURO

The role of the advisory group was to support WHO in planning and implementing the research prioritization process, and to provide input on the data analysis, interpretation and presentation of the results.

For more information on the process and functions of the TAG, as well as biographies of the TAG members, please see: **https://www.who.int/who-sti-research-priority-setting-technical-advisory-group**.

SURVEY 1 – Proposal of STI Research Priorities

TOP OF EACH PAGE: STI Research Priorities Survey

Page 1 of 8

This survey will provide an opportunity for you to suggest important research areas in the field of STIs. It is estimated to take approximately 20-30 minutes.

If you choose to participate, your responses will be confidential and anonymous. The survey will not be linked to any identifying information such as your name, email address or IP address.

If you wish to continue, please click “Next” below.

Otherwise, please close the webpage without clicking the “Next” button

NEXT

Page 2 of 8

Thank you for agreeing to participate in this survey

Participant characteristics

We would first like to obtain some information about you to help us understand the range of stakeholders participating in the survey.

1. What is(are) your main field(s) of expertise? (Select all that apply)
    - Sexual and reproductive health
    - Sexually transmitted infections (other than HIV)
    - HIV/AIDS
    - Obstetrics/gynecology
    - Infectious diseases
    - Primary care
    - Women’s health
    - Adolescent health
    - Key populations (e.g., MSM, sex workers, transgender persons, people who inject drugs, incarcerated populations)
    - Other, specify______

2. What is your primary occupation?
    - Policy maker
    - Programme manager
    - Researcher
    - Nurse
    - Doctor
    - Other health care provider, specify______
    - Academic/educator

Funder
Commercial (e.g. pharmacy, diagnostic manufacturers)
Other specify_____)

3. Please select the main type of employer or organization you work in.
   National or regional government
   Non-governmental organization
   Non-profit, specify_____
   Academic/research institution
   Hospital/clinic
   UN agency
   International organization (non-UN)
   Foundation
   Commercial/private
   Other, specify_______

4. The primary focus of your work is in which of the following WHO regions? (Select all that apply)
   African Region (AFR)
   Region of the Americas (AMR)
   South-East Asian Region (SEAR)
   European Region (EUR)
   Eastern Mediterranean Region (EMR)
   Western Pacific Region (WPR)
   Global

NOTE: the countries within each WHO region can be found at: https://www.who.int/countries

5. My work primarily focuses on the following country income level
   Low-income countries (LIC)
   Low-middle income countries (LMIC)
   Upper-middle income countries (UMIC)
   High income countries (HIC)

6. Number of years doing work related to STIs including HIV/AIDS: [ ] years

7. Age
   18-24 years
   25-34 years
   35-44 years
   45-54 years
   55-64 years
   65 years and over

8. What is your gender identity? (Select all that apply)

Woman
Man
No gender
Transgender woman
Transgender man
Non-binary
Not described above, specify_______
Prefer not to disclose/self-describe

NEXT

Page 3 of 8

Instructions

In the next section you will be asked to propose research areas that you consider most important to fill knowledge gaps related to STIs, which have not yet been adequately addressed. The proposed STI research areas should:

- Involve any STI pathogen, population, or setting you feel is important
- Generate knowledge within the next 2-8 years
- Focus on low and-middle- income countries (LMIC) or underserved areas or populations in high-income countries (HICs)

*NOTE: Research priorities related to HIV, hepatitis, HPV vaccination and cervical cancer prevention are being addressed elsewhere. Other research areas related to HPV may be raised.

You will be asked to propose up to 3-5 research areas for each of three categories of research:

1. Research to understand the extent of the problem and its predictors
2. Research to develop, design, and evaluate new interventions
3. Research to implement existing STI interventions

In developing your research area statements, please consider there will be a follow-up survey to rank research areas according to the following 4 criteria (as outlined on the process information sheet):

1. Public health relevance: The emerging intervention is likely to substantially improve health.
2. Research feasibility*:* It will be possible to design an ethically sound and implementable research study to address the proposed research area.
3. Programme feasibility: The research results can be translated into a deliverable and affordable public health intervention.
4. Equity value: Addressing the research area can facilitate interventions that reduce population inequities.

NEXT

Page 4 of 8

Priority research areas

Research to understand the extent of the problem and its predictors (e.g., epidemiology, risk factors, consequences, disease burden).

This research seeks to understand the STI and its outcomes, including the epidemiology of infection and disease, risk factors, and consequences. Consequences can be health, social, and economic outcomes.

Example: Estimate the prevalence of "x pathogen" infection among "y groups" in LMICs.

Example: Evaluate the burden of "x disease pathology" related to "y pathogen" in multiple settings.

9. Please propose up to 3-5 priority STI research areas below related to understanding the problem and predictors

When writing each research statement, please specify the relevant pathogen(s), population(s), and/or setting(s), if applicable.

9.1 Research priority #1

9.2 Research priority #2

9.3 Research priority #3

9.4 Research priority #4

9.5 Research priority #5

NEXT

Page 5 of 8

Priority research areas

Research to develop, design, and evaluate new interventions (e.g., technologies, products, programmes, clinical strategies).

This research seeks to discover, develop, and evaluate new interventions that will promote STI prevention, control and management. "Interventions" are not limited to technological innovations, but may also include programmatic interventions, clinical algorithms, treatment options, etc.

Example: Develop vaccines against "x pathogen"

Example: Evaluate "x clinical algorithm" for managing "y pathogen" among "z subpopulation"

10. Please propose up to 3-5 priority STI research areas below related to developing, designing, and evaluating new interventions

When writing each research statement, please specify the relevant pathogen(s), population(s), and/or setting(s), if applicable.

10.1 Research priority #1

10.2 Research priority #2

10.3 Research priority #3

10.4 Research priority #4

10.5 Research priority #5

NEXT

Page 6 of 8

Priority research areas

Research to implement existing STI interventions (e.g., integration, scale-up, policy, health economics).

This research seeks to gain knowledge and experience on implementing and scaling up existing interventions. This may include evaluation of the acceptability, feasibility, cost, cost-effectiveness, and coverage of an intervention or programme.

Example: Evaluate the acceptability and feasibility of scaling up "x intervention" for "y population" in LMICs.

Example: Evaluate the cost-effectiveness of implementing "x intervention" for STIs in "y setting".

11. Please propose up to 3-5 priority STI research areas below related to evaluating implementation of existing interventions.

When writing each research statement, please specify the relevant pathogen(s), population(s), and/or setting(s), if applicable.

11.1 Research priority #1

11.2 Research priority #2

11.3 Research priority #3

11.4 Research priority #4

11.5 Research priority #5

NEXT

Page 7 of 8

12. Please list any additional priority STI research areas that have not been identified or addressed in the above questions.

When writing each research statement, please specify the relevant pathogen(s), population(s), and/or setting(s), if applicable.

12.1 Research priority # 1

12.2 Research priority # 2

12.3 Research priority # 3

12.4 Research priority # 4

12.5 Research priority # 5

If you have any other comments or suggestions, please let us know in the box below

NEXT

Page 8 of 8

Your final survey responses will be submitted once you click the “Submit” button below.

Your responses will help develop a list of research areas that will be ranked according to pre-defined criteria in a follow-up survey.

If you would like to be contacted directly for the follow-up survey, please refer to email invitation for the current survey. It contains a link to provide your email address in a separate database.

You can also copy the link here https://sti-priority-test.mystudy.me/email and paste it into your browser after clicking on the “Submit” button below.

SUBMIT

SURVEY 2 – Scoring of STI Research Priorities

Page 1

STI Research Priorities Scoring Survey

Welcome to the STI Research Priorities Scoring Survey. This survey presents a series of proposed STI research areas. You will be asked to score each research area based on how strongly you agree with each of the following four criteria:

1. Public health relevance: The intervention emerging from the research is likely to substantially improve health.
2. Research feasibility*:* It will be possible to design an ethically sound and implementable research study to address the proposed research area.
3. Programme feasibility: The research results can be translated into a deliverable and affordable public health intervention.
4. Equity value: Addressing the research area can facilitate interventions that reduce population inequities.

If you choose to participate, your responses will be confidential and anonymous. The survey will not be linked to any identifying information such as your name, email address or IP address.

If you wish to continue, please click below.

Page 2

Thank you for agreeing to participate in this survey.

Participant characteristics

We would first like to obtain some information about you to help us understand the range of stakeholders participating in the survey.

13. What is(are) your main field(s) of expertise? (Select all that apply)
    - Sexual and reproductive health
    - Sexually transmitted infections (other than HIV)
    - HIV/AIDS
    - Obstetrics/gynecology
    - Infectious diseases
    - Primary care
    - Women's health
    - Adolescent health
    - Key populations (e.g., MSM, sex workers, transgender persons, people who inject drugs, incarcerated populations)
    - Other, specify______

14. What is your primary occupation?
    - Policy maker
    - Programme manager
    - Researcher
    - Nurse
    - Doctor
    - Other health care provider, specify______
    - Academic/educator
    - Funder
    - Commercial (e.g., pharmacy, diagnostic manufacturers)
    - Other specify_____)

15. Please select the main type of employer or organization you work in.
    - National or regional government
    - Non-governmental organization
    - Non-profit, specify_____
    - Academic/research institution
    - Hospital/clinic
    - UN agency
    - International organization (non-UN)
    - Foundation
    - Commercial/private
    - Other, specify_______

16. The primary focus of your work is in which of the following WHO regions? (Select all that apply)
    - African Region (AFR)
    - Region of the Americas (AMR)
    - South-East Asian Region (SEAR)
    - European Region (EUR)
    - Eastern Mediterranean Region (EMR)
    - Western Pacific Region (WPR)
    - Global

NOTE: the countries within each WHO region can be found at: https://www.who.int/countries

17. My work primarily focuses on the following country income level (select all that apply)
    - Low-income countries (LIC)
    - Low-middle income countries (LMIC)
    - Upper-middle income countries (UMIC)
    - High income countries (HIC)

18. Number of years doing work related to STIs including HIV/AIDS: [ ] years

19. Age

    18-24 years
    25-34 years
    35-44 years
    45-54 years
    55-64 years
    65+ years

20. What is your gender identity? (Select all that apply)

    Woman/female
    Man/male
    Transgender woman/female
    Transgender man/male
    Non-binary
    Not described above, specify_______
    Prefer not to disclose/self-describe

NEXT

Page 3

Instructions

1. Public health relevance: The intervention emerging from the research is likely to substantially improve health.
2. Research feasibility*:* It will be possible to design an ethically sound and implementable research study to address the proposed research area.
3. Programme feasibility: The research results can be translated into a deliverable and affordable public health intervention.
4. Equity value: Addressing the research area can facilitate interventions that reduce population inequities.

<u>Section 1: Research to understand the problem in a variety of populations and settings</u>

1. Estimate the prevalence and incidence of syphilis.
2. Estimate the prevalence and incidence of *N. gonorrhoeae* and *C. trachomatis* infections and coinfections at different anatomical sites.
3. Estimate the prevalence and incidence of genital HSV infections, and natural history.

4. Evaluate the burden of disease outcomes due to gonococcal and chlamydial infection (e.g., PID, infertility, adverse pregnancy outcomes.
5. Evaluate the burden of disease outcomes due to genital HSV infection (e.g., GUD, neonatal herpes.
6. Evaluate the burden of disease outcomes associated with syphilis.
7. Investigate whether *M. genitalium* infections lead to important disease outcomes and the natural history of infection.

8. Evaluate quality of life effects, health utility weights, disability weights, and societal costs associated with different STIs.
9. Evaluate the interactions between STIs and the vaginal microbiome.
10. Gain better understanding of STI transmission in populations using sexual network analysis, genomic epidemiology, and other innovative methods.
11. Evaluate the epidemiology and mechanisms of antimicrobial resistance and treatment failures for gonococcal, chlamydial, trichomonal, and *M. genitalium* infections, from different anatomical sites.

<u>Section 2: Research to develop and evaluate new interventions in a variety of populations and settings</u>

12. Develop and evaluate vaccines against herpes simplex virus (HSV).
13. Develop and evaluate vaccines against gonococcal infection (including group B meningitis vaccines).
14. Develop and evaluate vaccines against syphilis.
15. Develop and evaluate vaccines against chlamydial infection.

16. Develop and/or evaluate oral alternatives to benzathine penicillin for the treatment of syphilis during pregnancy (crossing placental/blood-brain barriers).
17. Develop better, ideally curative, treatment for HSV infection.
18. Develop or identify alternative therapeutics that can effectively treat Mycoplasma genitalium, including drug-resistant infections.
19. Develop new or identify existing therapeutics that can effectively treat gonococcal infection, including multi-drug resistant (MDR) infection, at multiple anatomic sites.
20. Develop or identify alternative therapeutics that can effectively treat trichomoniasis, including drug-resistant infections.

21. Develop and evaluate low-cost, rapid point-of-care diagnostic tests for gonococcal infection, chlamydial infection, or both.
22. Develop and evaluate low-cost, rapid point-of-care diagnostic tests that can distinguish active syphilis from latent or past infection.
23. Develop and evaluate low-cost, rapid point-of-care diagnostic tests for trichomonal infection.
24. Develop and evaluate low-cost, rapid point-of-care diagnostic tests for HSV infection.
25. Develop and evaluate low-cost, rapid point-of-care diagnostic tests for *M. genitalium* infection.
26. Develop and evaluate low-cost, rapid point-of-care diagnostic tests for antimicrobial resistance (AMR), specifically for gonorrhea and *M. genitalium.*

27. Design and/or evaluate improved tools or methods (e.g., biomarkers, imaging) for diagnosing pelvic inflammatory disease (PID).
28. Develop and evaluate multiplex platforms or other improved technologies for diagnosing etiologies of STI-related syndromes (e.g., vaginal discharge, urethral discharge, genital ulcer disease).
29. Develop improved diagnostic and therapeutic options for congenital syphilis, neurosyphilis, and other longer-term sequelae of syphilis (e.g., cardiovascular disease).
30. Design and evaluate multipurpose technologies to prevent STIs and pregnancy (with or without HIV prevention).

Section 3: Research to evaluate implementation of existing interventions (e.g., determine-acceptability, feasibility, effectiveness, and/or cost-effectiveness) in a variety of at-risk populations and settings

31. Evaluate the implementation of diagnostic testing for STI symptoms as opposed to syndromic management.
32. Evaluate the implementation of STI partner management, especially in LMICs.
33. Evaluate the implementation of rapid diagnostic tests to screen for syphilis.
34. Evaluate the implementation of rapid diagnostic tests to screen for gonorrhea, chlamydia, and trichomoniasis.
35. Evaluate the implementation of self-sampling or self-testing for STIs.

36. Evaluate pre- and post-exposure prophylactic strategies for STIs and implementation into programmes, including effects on AMR and the microbiome.
37. Evaluate whether screening and treatment for STIs reduces adverse pregnancy outcomes.
38. Design and evaluate strategies to reduce stigma and adverse psychosocial consequences associated with STI diagnoses (e.g., counseling, disclosure strategies).
39. Design and evaluate communication strategies, including social media and community-based approaches to increase STI awareness, prevention, and service engagement among populations at risk.
40. Assess the patterns, facilitators, and barriers of STI healthcare-seeking behavior, especially for adolescents, young people, and marginalized populations in LMICs.

Section 4: Sexual health-related research on mpox

41. Evaluate the risk and determinants of acquisition and transmission of monkeypox virus associated with different types of sexual contact, behavior, and mpox clinical presentations for a variety of populations and settings.
42. Investigate the spectrum and determinants of mpox clinical presentation, progression, severity, complications and sequelae, including site and dose of inoculum, type of sexual contact, STI/HIV status, gender, and pregnancy status.
43. Evaluate the duration and dynamics of monkeypox viral persistence and potential infectiousness in semen and other bodily fluids, shedding from mucosal or skin sites, and immune responses, according to population and immune status.
44. Evaluate the efficacy and/or effectiveness of smallpox/mpox vaccines against sexually acquired mpox and risk of reinfection or recurrence.
45. Evaluate the efficacy and/or effectiveness of antiviral treatments for sexually acquired mpox and associated factors (e.g., timing of treatment, drug levels in different body fluids, emerging risk of resistance to treatment).
46. Evaluate the barriers to prevention and care for mpox, experiences of stigma and discrimination, and effective risk communication and community engagement strategies in different contexts.

Table S1. Demographics of the respondents to the STI research priority scoring survey

	Survey respondents (n=289)	
Gender identity		
Woman/female	151	(53%)
Man/male	133	(46%)
Non-binary, more than one gender, or prefer not to self-describe	3	(1%)
Age group, years		
18-24	8	(3%)
25-34	35	(12%)
35-44	83	(29%)
45-54	70	(24%)
55-64	62	(22%)
65 and over	29	(10%)
WHO Region of primary focus of work		
African Region	37	(13%)
Region of the Americas	55	(19%)
South-East Asian Region	56	(20%)
European Region	30	(11%)
Eastern Mediterranean Region	22	(8%)
Western Pacific Region	24	(8%)
More than one WHO Region or global	61	(21%)
Country-income level of primary focus of work		
Low-income only	36	(13%)
Middle-income only	132	(46%)
High-income only	46	(16%)
Low-income and middle-income	42	(15%)
Low- or middle-income and high-income	31	(11%)
Number of years doing work related to STIs, including HIV	16	(1-43)
Primary occupation		
Researcher	73	(25%)
Academic/educator	34	(12%)
Health care provider	111	(38%)
Programme manager or policymaker	43	(15%)
Other field	28	(10%)
Main type of employer or organization		
Academic/research institution	87	(30%)
Hospital or clinic	46	(16%)
National or regional government	66	(23%)
Non-governmental or nonprofit organization	31	(11%)
International organization	30	(10%)

Other (e.g., commercial or private institution, foundation)	29	(10%)
Main field(s) of expertise (could choose more than one)		
STIs other than HIV	201	(70%)
HIV/AIDS	150	(52%)
Infectious diseases	109	(38%)
Sexual and reproductive health	106	(37%)
Women's health or obstetrics/gynecology	64	(22%)
Adolescent health	39	(13%)
Primary care	36	(12%)
Key populations	102	(35%)
Other	18	(6%)

Data are n (%) or median (range), where % accounts for number of respondents with data available. Denominators for calculating % may vary slightly, reflecting missing values for some variables.

Figure S1. Priority STI research areas within the diagnosis domain, according to levels of stakeholder agreement and mean research priority score

		Public health relevance	Research feasibility	Programme feasibility	Equity value	Research priority score
**STI research areas**	n	% Strongly agree	% Strongly agree	% Strongly agree	% Strongly agree	Mean
Develop and evaluate low-cost, rapid point-of-care diagnostic tests for gonococcal infection, chlamydial infection, or both	258	76	62	65	64	91
Develop and evaluate low-cost, rapid point-of-care diagnostic tests that can distinguish active syphilis from latent or past infection	251	74	60	63	60	91
Evaluate the implementation of rapid diagnostic tests to screen for syphilis	248	70	65	63	57	91
Evaluate the implementation of rapid diagnostic tests to screen for gonorrhea, chlamydia, and trichomoniasis	252	68	60	53	59	90
Develop and evaluate low-cost, rapid point-of-care diagnostic tests for antimicrobial resistance (AMR), specifically for gonorrhea and *M. genitalium*	251	63	52	45	49	87
Evaluate the implementation of diagnostic testing for STI symptoms as opposed to syndromic management	252	63	56	48	49	87
Evaluate the implementation of self-sampling or self-testing for STIs	252	56	51	50	50	86
Develop and evaluate multiplex platforms or other improved technologies for diagnosing etiologies of STI-related syndromes (e.g., vaginal discharge, urethral discharge, genital ulcer disease)	249	55	47	41	42	85
Develop and evaluate low-cost, rapid point-of-care diagnostic tests for HSV infection	251	47	43	43	42	83
Develop and evaluate low-cost, rapid point-of-care diagnostic tests for trichomonal infection	251	42	38	36	35	82
Design and/or evaluate improved tools or methods (e.g., biomarkers, imaging) for diagnosing pelvic inflammatory disease (PID)	251	39	30	26	31	80
Develop and evaluate low-cost, rapid point-of-care diagnostic tests for *M. genitalium* infection	251	36	31	28	28	78

The research priority score reflects the mean summary score across all levels of agreement and across all 4 criteria adapted to a 100% scale.

- ≥55% Strongly agree
- 40-54% Strongly agree
- <40% Strongly agree

Figure S2. Priority STI research areas within the prevention domain, according to levels of stakeholder agreement and mean research priority score

		Public health relevance	Research feasibility	Programme feasibility	Equity value	Research priority score
**STI research areas**	n	% Strongly agree	% Strongly agree	% Strongly agree	% Strongly agree	Mean
Design and evaluate multipurpose technologies to prevent STIs and pregnancy (with or without HIV prevention)	248	61	48	47	51	87
Develop and evaluate vaccines against gonococcal infection (including group B meningitis vaccines)	253	65	51	49	52	87
Design and evaluate communication strategies, including social media and community-based approaches to increase STI awareness, prevention, and service engagement among populations at risk	249	57	50	49	50	86
Evaluate whether screening and treatment for STIs reduces adverse pregnancy outcomes	249	56	47	49	51	86
Develop and evaluate vaccines against herpes simplex virus (HSV)	256	67	45	46	51	86
Develop and evaluate vaccines against syphilis	252	63	42	48	51	85
Develop and evaluate vaccines against chlamydial infection	252	57	41	42	48	84
Evaluate pre- and post-exposure prophylactic strategies for STIs and implementation into programmes, including effects on AMR and the microbiome	250	53	40	38	36	83

** The research priority score reflects the mean summary score across all levels of agreement and across all 4 criteria adapted to a 100% scale.*

≥55% Strongly agree

40-54% Strongly agree

<40% Strongly agree

Figure S3. Priority STI research areas within the management domain, according to levels of stakeholder agreement and mean research priority score

STI research areas	n	Public health relevance<br>% Strongly agree	Research feasibility<br>% Strongly agree	Programme feasibility<br>% Strongly agree	Equity value<br>% Strongly agree	Research priority score<br>Mean
Develop new or identify existing therapeutics that can effectively treat gonococcal infection, including multi-drug resistant (MDR) infection, at multiple anatomic sites	249	67	55	55	51	89
Develop and/or evaluate oral alternatives to benzathine penicillin for the treatment of syphilis during pregnancy (crossing placental/blood-brain barriers)	255	61	42	48	52	86
Evaluate the implementation of STI partner management, especially in LMICs	251	57	39	38	50	85
Develop improved diagnostic and therapeutic options for congenital syphilis, neurosyphilis, and other longer-term sequelae of syphilis (e.g., cardiovascular disease)	250	50	41	40	45	85
Develop better, ideally curative, treatment for HSV infection	255	56	40	39	39	84
Design and evaluate strategies to reduce stigma and adverse psychosocial consequences associated with STI diagnoses (e.g., counseling, disclosure strategies)	246	49	39	37	45	84
Develop or identify alternative therapeutics that can effectively treat trichomoniasis, including drug-resistant infections	247	31	29	28	29	81
Develop or identify alternative therapeutics that can effectively treat *Mycoplasma genitalium* , including drug-resistant infections	247	30	25	22	25	79

** The research priority score reflects the mean summary score across all levels of agreement and across all 4 criteria adapted to a 100% scale.*

≥55% Strongly agree
40-54% Strongly agree
<40% Strongly agree

Figure S4. Priority STI research areas within the epidemiology domain, according to levels of stakeholder agreement and mean research priority score

		Public health relevance	Research feasibility	Programme feasibility	Equity value	Research priority score
**STI research areas**	n	% Strongly agree	% Strongly agree	% Strongly agree	% Strongly agree	Mean
Estimate the prevalence and incidence of syphilis	240	60	58	52	51	88
Assess the patterns, facilitators, and barriers of STI healthcare-seeking behavior, especially for adolescents, young people, and marginalized populations in LMICs	248	57	47	45	54	87
Evaluate the epidemiology and mechanisms of antimicrobial resistance and treatment failures for gonococcal, chlamydial, trichomonal, and *M. genitalium* infections, from different anatomical sites	232	59	49	42	44	86
Evaluate the burden of disease outcomes associated with syphilis	240	58	48	46	46	86
Estimate the prevalence and incidence of *N. gonorrhoeae* and *C. trachomatis* infections and coinfections at different anatomical sites	239	51	46	38	42	85
Evaluate the burden of disease outcomes due to gonococcal and chlamydial infection (e.g., PID, infertility, adverse pregnancy outcomes	239	55	45	37	44	85
Evaluate quality of life effects, health utility weights, disability weights, and societal costs associated with different STIs	235	43	38	33	42	82
Gain better understanding of STI transmission in populations using sexual network analysis, genomic epidemiology, and other innovative methods	232	43	37	30	32	81
Evaluate the burden of disease outcomes due to genital HSV infection (e.g., GUD, neonatal herpes	241	37	35	28	33	81
Estimate the prevalence and incidence of genital HSV infections, and natural history	239	38	37	30	30	80
Investigate whether *M. genitalium* infections lead to important disease outcomes and the natural history of infection	237	31	29	26	28	77
Evaluate the interactions between STIs and the vaginal microbiome	233	28	29	20	22	76

** The research priority score reflects the mean summary score across all levels of agreement and across all 4 criteria adapted to a 100% scale.*

- ≥55% Strongly agree
- 40-54% Strongly agree
- <40% Strongly agree

Table S2. Diagnosis domain: STI research areas ranked by research priority score (RPS), overall and by WHO Region and by country-income level

		WHO Regions							Income levels		
**STI research priority areas**	**All respondents** Rank (RPS) n=289	**AFR** Rank (RPS) n=37	**AMR** Rank (RPS) n=55	**SEAR** Rank (RPS) n=56	**EUR** Rank (RPS) n=30	**EMR** Rank (RPS) n=22	**WPR** Rank (RPS) n=24	**>1 Region or global** Rank (RPS) n=61	**LMICs** Rank (RPS) n=212	**HICs** Rank (RPS) n=46	**Both LMICs/HICs** Rank (RPS) n=31
Develop and evaluate low-cost, rapid point-of-care diagnostic tests for gonococcal infection, chlamydial infection, or both	1 (91)	1 (92)	1 (95)	5 (89)	2 (90)	2 (84)	4 (93)	1 (92)	1 (91)	1 (91)	1 (94)
Develop and evaluate low-cost, rapid point-of-care diagnostic tests that can distinguish active syphilis from latent or past infection	2 (91)	3 (90)	4 (92)	2 (90)	1 (91)	4 (84)	3 (94)	2 (91)	3 (91)	3 (90)	2 (91)
Evaluate the implementation of rapid diagnostic tests to screen for syphilis	3 (91)	5 (88)	2 (94)	1 (91)	3 (90)	3 (84)	2 (95)	3 (90)	2 (91)	2 (91)	5 (89)
Evaluate the implementation of rapid diagnostic tests to screen for gonorrhea, chlamydia, and trichomoniasis	4 (90)	2 (90)	3 (93)	3 (90)	4 (90)	5 (83)	1 (95)	4 (88)	4 (90)	4 (90)	4 ((89)
Develop and evaluate low-cost, rapid point-of-care diagnostic tests for antimicrobial resistance (AMR), specifically for gonorrhea and *M. genitalium*	5 (87)	6 (88)	7 (87)	8 (85)	5 (87)	1 (85)	6 (91)	6 (86)	6 (87)	6 (87)	3 (89)
Evaluate the implementation of diagnostic testing for STI symptoms as opposed to syndromic management	6 (87)	4 (90)	6 (88)	4 (89)	6 (87)	12 (81)	7 (89)	7 (84)	5 (88)	8 (85)	7 (85)
Evaluate the implementation of self-sampling or self-testing for STIs	7 (86)	8 (85)	5 (90)	11 (80)	8 (85)	6 (82)	5 (92)	5 (87)	7 (86)	5 (88)	6 (86)
Develop and evaluate multiplex platforms or other improved technologies for diagnosing etiologies of STI-related syndromes (e.g., vaginal discharge, urethral discharge, genital ulcer disease)	8 (85)	7 (86)	8 (87)	7 (87)	7 (85)	7 (82)	8 (84)	9 (82)	8 (85)	7 (86)	10 (80)
Develop and evaluate low-cost, rapid point-of-care diagnostic tests for HSV infection	9 (83)	9 (83)	10 (86)	9 (85)	9 (85)	10 (81)	12 (75)	8 (83)	9 (85)	10 (78)	9 (81)
Develop and evaluate low-cost, rapid point-of-care diagnostic tests for trichomonal infection	10 (82)	11 (81)	9 (86)	6 (87)	10 (82)	8 (82)	9 (77)	11 (77)	10 (82)	9 (81)	8 (84)
Design and/or evaluate improved tools or methods (e.g., biomarkers, imaging) for diagnosing pelvic inflammatory disease (PID)	11 (80)	10 (81)	12 (81)	10 (82)	11 (77)	11 (81)	10 (76)	10 (79)	11 (81)	11 (77)	12 (75)
Develop and evaluate low-cost, rapid point-of-care diagnostic tests for *M. genitalium* infection	12 (78)	12 (75)	11 (83)	12 (79)	12 (74)	9 (81)	11 (76)	12 (74)	12 (80)	12 (72)	11 (75)

AFR, WHO African Region; AMR, WHO Region of the Americas; SEAR, WHO South-East Asian Region; EUR, WHO European Region; EMR, WHO Eastern Mediterranean Region; WPR, WHO Western Pacific Region; LMICs, low- and middle-income countries; HICs, high-income countries

Table S3. Prevention domain: STI research areas ranked by research priority score (RPS), overall and by WHO Region and by country-income level

			WHO Regions								Income levels		
**STI research priority areas**	**All respondents** Rank (RPS) n=289		**AFR** Rank (RPS) n=37	**AMR** Rank (RPS) n=55	**SEAR** Rank (RPS) n=56	**EUR** Rank (RPS) n=30	**EMR** Rank (RPS) n=22	**WPR** Rank (RPS) n=24	**>1 Region or global** Rank (RPS) n=61		**LMICs** Rank (RPS) n=212	**HICs** Rank (RPS) n=46	**Both LMICs/HICs** Rank (RPS) n=31
Design and evaluate multipurpose technologies to prevent STIs and pregnancy (with or without HIV prevention)	1 (87)		1 (87)	2 (90)	2 (88)	5 (85)	3 (83)	5 (86)	5 (87)		1 (87)	5 (87)	6 (86)
Develop and evaluate vaccines against gonococcal infection (including group B meningitis vaccines)	2 (87)		2 (87)	3 (90)	6 (81)	3 (89)	6 (74)	1 (90)	1 (90)		5 (85)	1 (90)	1 (94)
Design and evaluate communication strategies, including social media and community-based approaches to increase STI awareness, prevention, and service engagement among populations at risk	3 (86)		8 (81)	8 (86)	1 (89)	2 (90)	1 (89)	2 (90)	7 (83)		2 (87)	6 (85)	5 (86)
Evaluate whether screening and treatment for STIs reduces adverse pregnancy outcomes	4 (86)		6 (84)	5 (89)	3 (87)	6 (85)	2 (89)	7 (84)	6 (84)		4 (86)	3 (87)	7 (84)
Develop and evaluate vaccines against herpes simplex virus (HSV)	5 (86)		5 (85)	4 (90)	4 (83)	1 (92)	8 (73)	6 (85)	3 (88)		3 (86)	7 (83)	2 (90)
Develop and evaluate vaccines against syphilis	6 (85)		3 (85)	1 (91)	8 (78)	4 (87)	5 (75)	4 (87)	2 (89)		6 (84)	2 (89)	3 (89)
Develop and evaluate vaccines against chlamydial infection	7 (84)		4 (85)	6 (88)	7 (80)	8 (84)	7 (73)	3 (87)	4 (87)		8 (83)	4 (87)	4 (87)
Evaluate pre- and post-exposure prophylactic strategies for STIs and implementation into programmes, including effects on AMR and the microbiome	8 (83)		7 (84)	7 (88)	5 (81)	7 (85)	4 (79)	8 (82)	8 (83)		7 (84)	8 (83)	8 (80)

AFR, WHO African Region; AMR, WHO Region of the Americas; SEAR, WHO South-East Asian Region; EUR, WHO European Region; EMR, WHO Eastern Mediterranean Region; WPR, WHO Western Pacific Region; LMICs, low- and middle-income countries; HICs, high-income countries

Table S4. Management domain: STI research areas ranked by research priority score (RPS), overall and by WHO Region and by country-income level

		WHO Regions							Income levels		
**STI research priority areas**	**All respondents** Rank (RPS) n=289	**AFR** Rank (RPS) n=37	**AMR** Rank (RPS) n=55	**SEAR** Rank (RPS) n=56	**EUR** Rank (RPS) n=30	**EMR** Rank (RPS) n=22	**WPR** Rank (RPS) n=24	**>1 Region or global** Rank (RPS) n=61	**LMICs** Rank (RPS) n=212	**HICs** Rank (RPS) n=46	**Both LMICs/HICs** Rank (RPS) n=31
Develop new or identify existing therapeutics that can effectively treat gonococcal infection, including multi-drug resistant (MDR) infection, at multiple anatomic sites	1 (89)	1 (92)	1 (92)	2 (88)	2 (86)	3 (83)	1 (89)	1 (92)	1 (89)	1 (87)	1 (92)
Develop and/or evaluate oral alternatives to benzathine penicillin for the treatment of syphilis during pregnancy (crossing placental/blood-brain barriers)	2 (86)	2 (87)	2 (90)	1 (89)	7 (79)	2 (85)	5 (83)	2 (87)	2 (87)	2 (83)	5 (82)
Evaluate the implementation of STI partner management, especially in LMICs	3 (85)	3 (87)	5 (86)	6 (86)	4 (84)	7 (79)	2 (87)	3 (87)	3 (86)	5 (82)	4 (83)
Develop improved diagnostic and therapeutic options for congenital syphilis, neurosyphilis, and other longer-term sequelae of syphilis (e.g., cardiovascular disease)	4 (85)	5 (82)	3 (89)	3 (87)	5 (82)	4 (82)	3 (85)	5 (82)	4 (86)	4 (82)	6 (82)
Develop better, ideally curative, treatment for HSV infection	5 (84)	4 (83)	4 (87)	4 (86)	1 (88)	5 (80)	8 (76)	4 (83)	5 (85)	6 (80)	3 (83)
Design and evaluate strategies to reduce stigma and adverse psychosocial consequences associated with STI diagnoses (e.g., counseling, disclosure strategies)	6 (84)	8 (78)	6 (85)	5 (86)	3 (85)	1 (85)	4 (83)	8 (78)	6 (83)	3 (83)	2 (87)
Develop or identify alternative therapeutics that can effectively treat trichomoniasis, including drug-resistant infections	7 (81)	6 (81)	7 (839	7 (84)	8 (79)	6 (80)	7 (79)	6 (81)	7 (81)	7 (78)	7 (79)
Develop or identify alternative therapeutics that can effectively treat *Mycoplasma genitalium*, including drug-resistant infections	8 (79)	7 (79)	8 (82)	8 (82)	6 (80)	8 (79)	8 (76)	7 (79)	8 (80)	8 (77)	8 (77)

AFR, WHO African Region; AMR, WHO Region of the Americas; SEAR, WHO South-East Asian Region; EUR, WHO European Region; EMR, WHO Eastern Mediterranean Region; WPR, WHO Western Pacific Region; LMICs, low- and middle-income countries; HICs, high-income countries

Table S5: Epidemiology domain: STI research areas ranked by research priority score (RPS), overall and by WHO Region and by country-income level

		WHO Regions							Income levels		
**STI research priority areas**	**All respondents** Rank (RPS) n=289	**AFR** Rank (RPS) n=37	**AMR** Rank (RPS) n=55	**SEAR** Rank (RPS) n=56	**EUR** Rank (RPS) n=30	**EMR** Rank (RPS) n=22	**WPR** Rank (RPS) n=24	**>1 Region or global** Rank (RPS) n=61	**LMICs** Rank (RPS) n=212	**HICs** Rank (RPS) n=46	**Both LMICs/HICs** Rank (RPS) n=31
Estimate the prevalence and incidence of syphilis	1 (88)	3 (86)	2 (90)	1 (90)	2 (86)	1 (87)	2 (90)	3 (86)	1 (88)	1 (88)	1 (91)
Assess the patterns, facilitators, and barriers of STI healthcare-seeking behavior, especially for adolescents, young people, and marginalized populations in LMICs	2 (87)	9 (81)	4 (88)	2 (87)	1 (88)	2 (84)	3 (90)	9 (81)	3 (86)	3 (86)	5 (89)
Evaluate the epidemiology and mechanisms of antimicrobial resistance and treatment failures for gonococcal, chlamydial, trichomonal, and *M. genitalium* infections, from different anatomical sites	3 (86)	1 (87)	3 (89)	5 (86)	4 (84)	4 (83)	1 (93)	1 (87)	2 (86)	2 (86)	2 (90)
Evaluate the burden of disease outcomes associated with syphilis	4 (86)	7 (82)	1 (90)	3 (87)	3 (86)	3 (84)	4 (89)	7 (82)	4 (86)	4 (86)	4 (89)
Estimate the prevalence and incidence of *N. gonorrhoeae* and *C. trachomatis* infections and coinfections at different anatomical sites	5 (85)	4 (86)	5 (88)	4 (86)	7 (83)	6 (81)	6 (86)	4 (86)	5 (85)	5 (85)	3 (90)
Evaluate the burden of disease outcomes due to gonococcal and chlamydial infection (e.g., PID, infertility, adverse pregnancy outcomes	6 (85)	2 (86)	6 (88)	6 (85)	6 (84)	5 (81)	5 (87)	2 (86)	6 (84)	6 (84)	6 (87)
Evaluate quality of life effects, health utility weights, disability weights, and societal costs associated with different STIs	7 (82)	11 (81)	7 (86)	10 (80)	5 (84)	9 (78)	7 (83)	11 (80)	7 (83)	7 (83)	10 (80)
Gain better understanding of STI transmission in populations using sexual network analysis, genomic epidemiology, and other innovative methods	8 (81)	5 (82)	10 (81)	7 (83)	10 (77)	7 (79)	8 (82)	5 (82)	8 (82)	8 (82)	9 (81)
Evaluate the burden of disease outcomes due to genital HSV infection (e.g., GUD, neonatal herpes)	9 (81)	8 (81)	8 (85)	11 (79)	8 (81)	8 (78)	11 (75)	8 (81)	10 (81)	10 (81)	8 (83)
Estimate the prevalence and incidence of genital HSV infections, and natural history	10 (80)	6 (82)	9 (81)	8 (82)	9 (79)	10 (78)	12 (74)	6 (82)	9 (81)	9 (81)	7 (85)
Investigate whether *M. genitalium* infections lead to important disease outcomes and the natural history of infection	11 (77)	10 (81)	12 (79)	12 (77)	11 (76)	11 (75)	9 (81)	10 (81)	11 (78)	11 (78)	11 (76)

Evaluate the interactions between STIs and the vaginal microbiome	12 (76)		12 (79)	11 (79)	9 (82)	12 (75)	12 (73)	10 (75)	12 (79)		12 (76)	12 (76)	12 (69)

AFR, WHO African Region; AMR, WHO Region of the Americas; SEAR, WHO South-East Asian Region; EUR, WHO European Region; EMR, WHO Eastern Mediterranean Region; WPR, WHO Western Pacific Region; LMICs, low- and middle-income countries; HICs, high-income countries

Note: Additional analyses related to stakeholder expertise and affiliated organizations were explored (data not shown), but these did not reveal any relevant differences.

Table S6. Research priorities related to sexually acquired mpox (monkeypox virus)

Research area	Mean research priority score (SD)
Evaluate the efficacy and/or effectiveness of smallpox/mpox vaccines against sexually acquired mpox and risk of reinfection or recurrence.	80 (17)
Evaluate the efficacy and/or effectiveness of antiviral treatments for sexually acquired mpox and associated factors (e.g., timing of treatment, drug levels in different body fluids, emerging risk of resistance to treatment).	78 (17)
Evaluate the barriers to prevention and care for mpox, experiences of stigma and discrimination, and effective risk communication and community engagement strategies in different contexts.	77 (18)
Investigate the spectrum and determinants of mpox clinical presentation, progression, severity, complications and sequelae, including site and dose of inoculum, type of sexual contact, STI/HIV status, gender, and pregnancy status.	76 (17)
Evaluate the duration and dynamics of monkeypox viral persistence and potential infectiousness in semen and other bodily fluids, shedding from mucosal or skin sites, and immune responses, according to population and immune status.	75 (18)
Evaluate the risk and determinants of acquisition and transmission of monkeypox virus associated with different types of sexual contact, behavior, and mpox clinical presentations for a variety of populations and settings.	75 (18)

SD, standard deviation
